# Sufficient dimension reduction on partially nonlinear index models with applications to medical costs analysis

**DOI:** 10.1371/journal.pone.0321796

**Published:** 2025-05-13

**Authors:** Xiaobing Zhao, Yufeng Xia, Xuan Xu

**Affiliations:** 1 School of Data Sciences, Zhejiang University of Finance and Economics, Hangzhou, Zhejiang Province, China; 2 Asset and Laboratory Management Office, Zhejiang University of Finance and Economics, Hangzhou, Zhejiang Province, China; Semnan University, IRAN, ISLAMIC REPUBLIC OF

## Abstract

Modeling medical costs is a crucial task in health economics, especially when high-dimensional covariates and nonlinear effects are present. In this study, we propose a partially nonlinear index model (PNIM) that integrates partially sufficient dimension reduction with a rapid instrumental variable pilot estimation method. Through simulations, we demonstrate that the proposed model excels at capturing significant nonlinear relationships. When applying the model to the Medical Expenditure Panel Survey (MEPS) dataset, we identify important nonlinear age effects on medical costs and highlight key factors such as hospitalization, cardiovascular diseases, and supplemental insurance coverage. These findings provide valuable insights for healthcare policy, including targeted interventions for specific age groups and enhanced management of chronic conditions. Overall, the proposed method offers a flexible and computationally efficient framework for analyzing complex medical cost data, with broad applicability in health economics.

## Introduction

Modeling medical costs is a central issue in health economics and insurance, particularly for identifying risk factors and informing cost-effective healthcare interventions. However, the presence of severe skewness, and complex nonlinear effects in medical cost data poses significant challenges for accurate modeling and analysis.

To address longitudinal medical cost data, various statistical methods have been developed. For example, marginal models describe relationships among repeated observations [1], Markov models analyze transitions between health states over time [2], and random effects models capture relationships between medical expenditures and terminal events such as death [3]. Recently, a growing number of models have been proposed to address high-dimensional covariates in medical cost data. Prominent examples include generalized partially linear models [4, 5], single-index models [6–8], and partially nonlinear single-index models [9–11]. These frameworks offer flexibility for analyzing skewed medical cost data but often assume a known link function, which reduces their adaptability in practical settings. Furthermore, studies relying on a subset of covariates may overlook critical information inherent in high-dimensional data.

To overcome the “curse of dimensionality," sufficient dimension reduction techniques have emerged as effective tools aiming to reduce the dimensionality of the data while retaining all relevant information for prediction or inference. Key methodological advancements include techniques such as minimum average variance estimation [12], inverse regression methods [13–15], and semiparametric approaches [16]. However, these methods can fail in the presence of redundant variables. To address this issue, Chiaromonte *et al*. (2002) proposed partial sufficient dimension reduction for discrete variables [17], and Feng *et al*. (2013) extended this idea to continuous variables through the Partial Discretization Expectation Estimation (PDEE) method [18]. Partial sufficient dimension reduction effectively eliminates redundancy while preserving essential information, making it particularly suitable for high-dimensional and complex datasets.

Following dimension reduction, additive models have been widely applied for post-reduction modeling. Notable methods include backfitting algorithms [19], average regression estimation [20], and fast instrumental variable pilot estimation [21, 22]. While computationally efficient, these approaches face challenges in capturing multivariate nonlinear relationships and ensuring theoretical consistency under high-dimensional conditions.

To address these limitations, this paper proposes a Partially Nonlinear Index Model (PNIM) that integrates partial sufficient dimension reduction with fast instrumental variable pilot estimation. Unlike most existing models, the PNIM does not assume a known link function, providing greater flexibility in capturing complex relationships. By combining partial sufficient dimension reduction with fast instrumental variable pilot estimation, our method effectively handles high-dimensional covariates while maintaining computational efficiency. Simulation studies and real-world applications demonstrate the proposed model’s superior performance in identifying structural dimensions, accurately estimating relationships, and addressing challenges in medical cost analysis.

The remainder of this paper is organized as follows: Section 2 introduces the model framework; Section 3 outlines the estimation procedure; Section 4 discusses asymptotic properties; Section 5 presents simulation results; Section 6 applies the model to MEPS data; and Section 7 concludes with key findings and policy implications.

## Model Specification

Suppose that *Y* is a response variable and *X* is a *p*–dimensional covariate vector. The variation of the response can be explained by covariates with the general function $\psi (X)=\psi (X_{1},\ldots ,X_{p})$. Additionally, there may exist some nonlinear covariate effect, e.g., age on medical costs [4, 23]. Combining these two parts, we can construct the model below:

$$Y_{i}=\alpha +\psi (X_{i,1},\ldots ,X_{i,p})+f(T_{i})+\varepsilon_{i}.$$ (1)

However, ψ(·) is difficult to estimate accurately and challenging to implement in practice due to the “curse of dimensionality" when *p* is large [24]. To reduce dimensionality while explaining the variation in the response, we use an index model. Specifically, we aim to find q≤p such that ψ(·) can be approximated by a lower-dimensional function $g(X_{i}^{⊤}\beta_{1},\ldots ,X_{i}^{⊤}\beta_{q})$. Thus, our partially nonlinear index model (PNIM) is:

$$Y_{i}=\alpha +g(X_{i}^{⊤}\beta_{1},\ldots ,X_{i}^{⊤}\beta_{q})+f(T_{i})+\varepsilon_{i},\;q\leq p,$$ (2)

where α is the regression intercept, $X_{i}=(X_{i,1},\ldots ,X_{i,p}{)}^{⊤}\in R^{p}$ is a *p*-dimensional covariate vector, $T_{i}\in R^{1}$ is another covariate of interest which has a nonlinear effect on the outcome, $\beta_{j}=(\beta_{1j},\ldots ,\beta_{pj}{)}^{⊤}\in R^{p}$ for j=1,…,q, and g(·) is an unspecified *q*–variate regression function. We assume error term $\varepsilon_{i}$ to be independently identically distributed with mean 0 and finite variance $\sigma^{2}>0$, and $\left(X_{i},T_{i}\right)$ is independent of $\varepsilon_{i}$. Ideally, if $g(X_{i}^{⊤}\beta_{1},\ldots ,X_{i}^{⊤}\beta_{q})=\psi (X_{1},\ldots ,X_{p})$, we achieve *sufficient dimension reduction*, as extensively discussed in literature [25].

Model (2) is a very general form, including many existing semiparametric and nonparametric models as special cases. For instance, model (2) reduces to a nonparametric model if g(·)=0 [26]. If the index function g(x)=x and *q* = 1, model (2) becomes a partially linear model discussed by [27]. When the nonlinear function f(·)=0, model (2) reduces to a multi-index model, and the single-index model as its special case with *q* = 1 [28].

**Remark 1:** In model (2), g(·) and f(·) are unspecified functions to be estimated, $\beta \in R^{p}$ is an unknown parameter vector. For identifiability, as is common in additive models, we assume that E[g(X)]=E[f(T)]=0 (see Fan *et al*. (1998) [20]). Unlike Xia (2008) [24], we impose no restrictions on the parameter $B=(\beta_{1},\ldots ,\beta_{q})$ when applying the partial sufficient dimension reduction using the sliced inverse regression method [13], as this approach does not require a specific form of the regression function.

## Estimation for PNIM

We employ a two-step procedure for estimation in model (2). In the first step, we search for a lower *q*–dimensional covariate vector using partially sufficient dimension reduction [18]. Once we obtain the estimated basis direction $B=(\beta_{1},\ldots ,\beta_{q})$ and the structural dimension *q*, the second step involves estimating the nonparametric component f(·) via the fast instrumental variable pilot estimation [21], which is based on the standard additive model framework.

### Partial Sufficient Dimension Reduction

Dimension reduction plays a crucial role in addressing high-dimensional covariates in regression analysis. Traditional methods, such as minimum mean variance estimation [13], inverse regression methods [29, 30], and semiparametric estimation [25], provide effective tools for reducing dimensionality. However, these methods often fail to account for redundant variables in high-dimensional settings.

To address this limitation, we employ partial sufficient dimension reduction (PSDR), which extends the concept of sufficient dimension reduction by eliminating redundancy while preserving essential structural information. Specifically, we introduce the partial central subspace ${𝒮}_{Y|X}^{\left(T\right)}$, defined as the low-dimensional subspace of *X* that retains all information about *Y*, conditioned on *T*. Formally, ${𝒮}_{Y|X}^{\left(T\right)}$ satisfies:

$$Y⟂X|(P_{\left(𝒮\right)}X,T),$$ (3)

where ⟂ denotes statistical independence, and $P_{\left(𝒮\right)}$ is the orthogonal projection operator onto subspace 𝒮 in the standard inner product space. This implies that *Y* is independent of *X* given the projection $P_{\left(𝒮\right)}X$ and *T*.

The concept of the partial central subspace was first introduced by Chiaromonte *et al*. (2002) [17] for categorical variables *T*. However, when *T* is continuous, directly identifying ${𝒮}_{Y|X}^{\left(T\right)}$ becomes challenging due to the complexity of conditional distributions. To overcome this difficulty, we adopt the Partial Discretization Expectation Estimation (PDEE) method proposed by Feng *et al*., (2013) [18], which simplifies the estimation process through discretization.The PDEE method consists of two main steps: 1. Partial discretization. The continous variable *T* is transformed into a set of dichotomous variables. This step simplifies the estimation process by creating discrete strata based on *T*, making it easier to compute conditional expectations. 2. Subspace estimation. Using the transformed variables and the original predictors *X*, the partial central subspace $S_{Y|X}^{\left(T\right)}$ is estimated by finding the basis directions that retain the most information about *Y* . The specific steps of the operation are as follows:

**Step 1: Partial Discretization**

Discretize the continuous variable *T* into a set of binary variables. Let $T(s)=I_{\left\{T\leq s\right\}}$, where *s* is a sample observation value from a random variable *S* with support ${ℛ}_{S}^{1}$, and *I*_*A*_ denotes the indicator function of event *A*. This creates two sample subsets:

- $\{Y_{j},X_{j}{\}}_{T(s)=1}$, with sample size *n*_1_, corresponding to T(s)=1,
- $\{Y_{j},X_{j}{\}}_{T(s)=0}$, with sample size *n*_2_, corresponding to T(s)=0.

**Step 2: Subspace Estimation**

Let ${𝒮}_{Y|X}^{\left(T(s)\right)}$ denote the partial central subspace for Y⟂(X,T(s)). To estimate ${𝒮}_{Y|X}^{\left(T\right)}$, we define M(s) as a p×p positive semidefinite matrix, for any given *s*, there exists Span$\left\{M\left(s\right)\right\}=S_{Y|X}^{\left(T\left(s\right)\right)}$ and Span$\left\{M\right\}=S_{Y|X}^{\left(T\right)}$, where M=E{M(S)}. The estimating procedures for *M*(*s*) and *M* are detailed as follows:

- **Step 2-A:** For any fixed $s\in R_{S}^{1}$, we can gain *M*_*n*_(*s*), a consistent estimate of *M*(*s*), using available partial dimension reduction methods such as the partial sliced inverse regression (SIR) estimation [17], partial sliced average variance estimation (SAVE) [31], or partial directional regression (DR) [14].
  In this subsection, we utilize SIR method apply on the samples $\{Y_{j},X_{j}{\}}_{T(s)=1}$ and $\{Y_{j},X_{j}{\}}_{T(s)=0}$ to obtain the estimates of the basis $B_{1}=\left(\beta_{11},\ldots ,\beta_{1q_{1}}\right)$ and $B_{2}=\left(\beta_{21},\ldots ,\beta_{2q_{2}}\right)$, respectively. Then we have a p×p matrix $M_{n}\left(s\right)={\hat{B}}_{s}{\hat{B}}_{s}^{⊤}$, where ${\hat{B}}_{s}=\left({\hat{B}}_{1},{\hat{B}}_{2}\right)$.
- **Step 2-B:** *M*, the expected value of *M*(*s*), can be calculated by considering all possible values of *s*. Here, we choose an easy way where *s* is an independent copy of *T*. Let $\left\{s_{1},\ldots ,s_{l}\right\}$ be a set of *l* independent copies of *T*. For each *s*_*i*_, we gain *M*(*s*_*i*_), then *M* can be estimated by
  $$M=\underset{l\to \infty }{\lim}\frac{1}{l}\sum_{i=1}^{l}M\left(s_{i}\right)=E\left(M\left(S\right)\right).$$ (4)
- **Step 2-C:** Perform the spectral decomposition of *M*. Arrange the obtained feature vectors in descending order base on their eigenvalues$\left\{\gamma_{1},\gamma_{2},\cdots ,\gamma_{p}\right\}$. Assuming the structural dimension of the partially reduced subspace is *d*, then $span\left\{\gamma_{1},\gamma_{2},\cdots ,\gamma_{d}\right\}$ is the the estimate of the partially central subspace.

Existing methods in literature can be used to determine the structural dimension *d*, such as the sequential test method and the weighted sequential test method [13, 32]. In this paper, we apply the modified BIC-type criterion of Feng *et al*. (2013) [18] to estimate the structural dimension:

$$\hat{d}=\text{arg}\max_{k=1,2,\ldots ,p}\left(\frac{n\sum_{j=1}^{k}(\log({\hat{\lambda }}_{j}+1)-{\hat{\lambda }}_{j})}{2\sum_{j=1}^{p}(\log({\hat{\lambda }}_{j}+1)-{\hat{\lambda }}_{j})}-2C_{n}\times \frac{k(k+1)/2}{p}\right),$$ (5)

where ${\hat{\lambda }}_{1},\ldots ,{\hat{\lambda }}_{p}$ are the eigenvalues of matrix *M*_*n*_, k(k+1)/2 is the number of free parameters, and *C*_*n*_ is a penalty constant. In our simulation studies, we will follow Feng *et al*. (2013) [18] to choose $C_{n}=n^{1/3}p^{2/3}$.

### Fast instrumental variable pilot estimation

One can easily find that model (2) simplifies to a standard additive model if the basis direction $B=\left(\beta_{1},\cdots ,\beta_{q}\right)$ and the structural dimension *q* of dimension reduction subspace are estimated. We will discuss the estimation methods of the index function g(·) and the nonlinear function f(·) in the additive model as follows:

$$Y_{i}=g(Z_{i,1},\ldots ,Z_{i,q})+f(T_{i})+\alpha +\varepsilon_{i},$$ (6)

where $Z_{i,k}=X_{i}^{⊤}{\hat{\beta }}_{k}$ for k=1,…,q, and $({\hat{\beta }}_{1},\ldots ,{\hat{\beta }}_{q}{)}^{⊤}$ is an estimator of $(\beta_{1},\ldots ,\beta_{q}{)}^{⊤}$ shown in Subsection 3.1. For the standard additive model (6), average regression estimation is a popular consideration, such as average regression surface [20, 33], and modified average regression estimation [22]. In this paper, we extend the modified average regression method of Cheng *et al*. (2011) [22] to our models including a univariate index function or a multiple index function.

Model (6) can be rewritten as

$$Y_{i}=\tilde{\alpha }+\tilde{g}\left(Z_{i}\right)+\tilde{f}\left(T_{i}\right)+\varepsilon_{i},$$ (7)

where $\tilde{g}(Z)=g(Z)-E[g(Z)]$, $\tilde{f}(T)=f(T)-E[f(T)]$, and $\tilde{\alpha }=\alpha +E[g(Z)]+E[f(T)]$.

We can estimate the multivariate function $\tilde{g}(\cdot )$ and the univariate function $\tilde{f}(\cdot )$ using a method similar to Cheng *et al*. (2011) [22]. First, we define

$$E\left(Y|Z=z,T=t\right)=\tilde{\alpha }+\tilde{g}\left(z\right)+\tilde{f}\left(t\right)\equiv m\left(z,t\right).$$ (8)

Let $f_{Z}\left(z\right)$ and $f_{T}\left(t\right)$ be the density functions of *Z* and *T*, respectively, and the joint density function of (*Z*,*T*) is denoted by *f*(*z*,*t*). We further define a joint function

$$\Phi (z,t)=f_{Z}(z)f_{T}(t)/f(z,t).$$ (9)

One can easily find that this function has the following two attractive characteristics:

$$E[\Phi (Z,T)|Z=z]=1,\;E[\Phi (Z,T)\tilde{f}(T)|Z=z]=0.$$ (10)

Multiplying each side of (7) by Φ(Z,T), and further taking the expectation conditional on *Z* = *z*, we can remove the other nonparametric component f(·):

$$E\left(\Phi \left(Z,T\right)Y|Z=z\right)\equiv {\tilde{g}}^{*}\left(z\right)=\tilde{\alpha }+\tilde{g}\left(z\right).$$ (11)

This implies that an estimator of $\tilde{g}\left(z\right)$ can be obtained through the following equation

$$\tilde{g}\left(z\right)={\tilde{g}}^{*}\left(z\right)-\tilde{\alpha },$$ (12)

where the estimators of ${\tilde{g}}^{*}\left(z\right)$ and $\tilde{\alpha }$ are provided in (13) and (14) below. This estimator of $\tilde{g}\left(z\right)$ is a *modified average regression estimator*.

In the next step, we will give the estimators of ${\tilde{g}}^{*}\left(z\right)$ and $\tilde{\alpha }$, respectively. For the independent and identically distributed sample $\left(Y_{i},Z_{i},T_{i}\right),i=1,\ldots ,n$, we have $\tilde{\alpha }=E\left(Y|Z,T\right)=W(Z,T)$ from the identifiability condition and (7). Define $U=(Z^{⊤},T{)}^{⊤}$, thus we can estimate $\tilde{\alpha }$ by

$$\hat{\tilde{\alpha }}={\left(n^{2}\;h\right)}^{-1}\sum_{i=1}^{n}\sum_{j=1}^{n}K_{h_{0}}(U_{i},U_{j})Y_{i}=\bar{Y},$$ (13)

where $K_{h_{0}}\left(U_{i},u\right)={h_{0}}^{-(q+1)}\prod_{l=1}^{q+1}K\left(\frac{U_{i,l}-u_{l}}{h_{0}}\right)$, $U_{i}=(Z_{i,1},\ldots ,Z_{i,q},T_{i}{)}^{⊤}$, and $u=(u_{1},\ldots ,u_{q},u_{q+1}{)}^{⊤}$. The parameter *h*_0_ denotes a bandwidth (with the same definition for *h*_1_-*h*_6_ in the following). K(·) is a univariate kernel function. The choice of bandwidth *h* is critical in determining the smoothing level of the kernel estimator. In this work, we select *h* using cross-validation(CV), following the approach described by Cheng *et al*., 2011) [22], which aims to minimize the asymptotic mean squared error (AMSE). For The kernel function, we employ the standard normal density. This kernel is widely used due to its smoothness and well-behaved properties, which making it particularly effective for capturing the underlying structure of the data (see Cheng *et al*., 2011) [22]).

Following the similar results of Kim *et al*. (1999) [21], we can obtain an estimator of ${\tilde{g}}^{*}(\cdot )$ as follows:

$$\hat{\tilde{g^{*}}}\left(z\right)=n^{-1}\sum_{i=1}^{n}K_{h_{1}}(Z_{i},z)\frac{{\hat{f}}_{T}(T_{i})}{\hat{f}(Z_{i},T_{i})}Y_{i},$$ (14)

where $K_{h_{1}}\left(Z_{i},z\right)={h_{1}}^{-q}\prod_{l=1}^{q}K\left(\frac{Z_{i,l}-z_{l}}{h_{1}}\right)$ with $z=(z_{1},\ldots ,z_{q}{)}^{⊤}$. ${\hat{f}}_{T}(t)$ and $\hat{f}(z,t)$ are kernel smoothers of the corresponding densities *f*_*T*_(*t*) and *f*(*z*,*t*) using the functions $K_{h_{2}}(T_{i},t)=K\left(\frac{T_{i}-t}{h_{2}}\right)$ and $K_{h_{3}}(U_{i},u)={h_{3}}^{-(q+1)}\prod_{l=1}^{q+1}K\left(\frac{U_{i,l}-u_{l}}{h_{3}}\right)$, respectively.

Similarly, we can give an estimator of $\tilde{f}(t)$ as follows:

$$\hat{\tilde{f}}(t)=\hat{\tilde{f^{*}}}(t)-\hat{\tilde{\alpha }},$$ (15)

with $\hat{\tilde{\alpha }}$ given in (13). $\hat{\tilde{f^{*}}}(t)$ can be similarly given below

$$\hat{\tilde{f^{*}}}\left(t\right)=n^{-1}\sum_{i=1}^{n}K_{h_{4}}\left(T_{i},t\right)\frac{{\hat{f}}_{Z}\left(Z_{i}\right)}{\hat{f}\left(Z_{i},T_{i}\right)}Y_{i},$$ (16)

where $K_{h_{4}}(T_{i},t)=K\left(\frac{T_{i}-t}{h_{4}}\right)$. ${\hat{f}}_{Z}(z)$ and $\hat{f}(z,t)$ are the kernel smoothers of the corresponding densities *f*_*Z*_(*z*) and *f*(*z*,*t*), which are defined similarly in (14).

**Remark 2:** The modified average regression estimation proposed by Cheng *et al*. (2011) [22] extends the *fast instrumental variable pilot estimation* method of Kim *et al*. (1999) [21] as a practical alternative for the *quantile regression additive model*. The key difference between the two methods lies in the treatment of the response variable: Cheng *et al*. (2011) [22] replace the observed *Y* in Kim *et al*. (1999) [21] with the estimated W(z,t) (see Equations 13, (14), and (16)).

### Oracle efficient estimator

The variance of the estimator in the first step might be inflated according to the asymptotic properties of kim *et al*. (1999) [21]. Therefore, an oracle efficient estimate of model (7) is introduced herein, derived similar to Linton *et al*. (1997) [34] and kim *et al*. (1999) [21]. We show that it is asymptotically normally distributed, with the same variance as if the other additive components were known. The oracle estimate is obtained through the sequential fitting of univariate locally polynomial regression for each of the additive components of (7), with the other additive components replaced by their estimators from the first step.

Now we can discuss the oracle estimator for ${\tilde{g}}^{*}(z)$ and ${\tilde{f}}^{*}(t)$. Assume that ${\tilde{g}}^{*}(z)$ and ${\tilde{f}}^{*}(t)$ are *d* times (d≥2) continuously differentiable in the neighborhood of *z*_0_ and *t*_0_, respectively. Denote $\Lambda =(\lambda_{1},\ldots ,\lambda_{q})$ with nonnegative integers $\lambda_{i}$, i=1,2,…,q, $\left|\lambda \right|=\sum \lambda_{i}$, and $v^{\lambda }=\prod v_{i}^{\lambda_{i}},\left(\lambda \in \Lambda \;\;\text{and}\;v\in R^{q}\right)$. We have the local linear approximation of ${\tilde{g}}^{*}(z)$ near a fixed point *z*_0_ as follows:

$${\tilde{g}}^{*}(z)\approx {\tilde{g}}^{*}(z_{0})+\sum_{\lambda \in \Lambda }{{\left(\beta_{\lambda }^{z_{0}}\right)}^{⊤}h^{-\left|\lambda \right|}\left(z-z_{0}\right)}^{\lambda },$$ (17)

where *z* lies in a neighborhood of *z*_0_, and $\beta_{\lambda }^{z}=h^{-|\lambda |}\partial^{\lambda }\tilde{g}(z)/z_{1}^{\lambda_{1}},\ldots ,z_{q}^{\lambda_{q}}$.

Furthermore, define

$$Y_{i,-\tilde{g}}^{*}=Y_{i}+\bar{Y}-{\hat{\tilde{g}}}^{*}(z_{i}),\;Y_{i,-\tilde{f}}^{*}=Y_{i}-\bar{Y}-{\hat{\tilde{f}}}^{*}(t_{i}),$$ (18)

where ${\hat{\tilde{g}}}^{*}(z_{i})$ and ${\hat{\tilde{f}}}^{*}(t_{i})$ are the first step estimators defined in (14) and (16), respectively. Then, the oracle efficient estimator of ${\tilde{g}}^{*}(z)$ and ${\tilde{f}}^{*}(t)$, denoted as ${\tilde{g}}_{e}^{*}(z)$ and ${\tilde{f}}_{e}^{*}(t)$, can be obtained by minimizing the following terms, respectively:

$$n^{-1}\sum_{i=1}^{n}{\left(Y_{i,-\tilde{f}}^{*}-{\tilde{g}}^{*}(z_{0})-\sum_{\lambda \in \Lambda ,|\lambda |>0}(\beta_{\lambda }^{z_{0}}{)}^{⊤}h_{5}^{-|\lambda |}(Z_{i}-z_{0}{)}^{\lambda }\right)}^{2}L_{h_{5}}^{1}(Z_{i},z_{0}),$$ (19)

and

$$n^{-1}\sum_{i=1}^{n}{\left(Y_{i,-\tilde{g}}^{*}-{\tilde{f}}^{*}(t_{0})-(\beta^{t_{0}})(T_{i}-t_{0})\right)}^{2}L_{h_{6}}^{2}(T_{i},t_{0}),$$ (20)

where the kernel function $L_{h_{5}}^{1}\left(Z_{i},z_{0}\right)$ is a *q*–dimensional kernel function, which can be written as $L_{h_{5}}^{1}\left(Z_{i},z_{0}\right)=h_{5}^{-q}\prod_{l=1}^{q}L\left(\frac{Z_{i,l}-z_{0,l}}{h_{5}}\right)$, and $L_{h_{6}}^{2}\left(T_{i},t_{0}\right)=L\left(\frac{T_{i}-t_{0}}{h_{6}}\right)$ is a 1–dimensional kernel function with L(·) being a univariate kernel function.

## Asymptotic Properties

The asymptotic properties of the estimated basis direction $B=\left(\beta_{1},\cdots ,\beta_{q}\right)$ and the structural dimension *q* of the partial sufficient dimension reduction can be derived in a manner similar to that in Feng *et al*. (2013) [18]. The asymptotic results of the estimators for the nonparametric components ${\tilde{g}}^{*}(\cdot )$ and ${\tilde{f}}^{*}(\cdot )$ can be established similarly by using the asymptotic results of Kim *et al*. (1999) [21]. Here, we focus on the asymptotic results ${\tilde{g}}^{*}(\cdot )$ and ${\tilde{f}}^{*}(\cdot )$.

To establish the asymptotic properties of the proposed estimators, we need some regularity conditions where kernel functions $K_{h_{1}}(z,t)$, $K_{h_{2}}(z)$ and $K_{h_{3}}(t)$ are used to estimate the joint density *f*_*Z*,*T*_(*z*,*t*), *f*_*Z*_(*z*) and *f*_*T*_(*t*), respectively. These conditions are similar to those of kim *et al*. (1999) [21], but with some modification for our setting.

**A1**: The kernel function K(·) defined in Section 2.3 is symmetric about zero and of order *d* with support in [–1,1], implying that $\int u^{j}K(u)du=0,j=1,\ldots ,d-1$. Furthermore, *K*(*u*) is assumed to be bounded and Lipschitz continuous, i.e., there exists a constant *c* such that |K(u)−K(v)|≤c|u−v| for any *u*,*v*.

**A2**: Function m(x,t)=E[Y|z,t]=g(z)+f(t) and joint density function of *f*_*Z*,*T*_(*z*,*t*) are *d*–times continuously differentiable in each direction, where d>(q−1)/2.

**A3**: The joint density function *f*_*Z*,*T*_(*z*,*t*) is bounded away from both zero and infinity within its compact support.

**A4**: Conditional variances $\sigma^{2}(t)=var(Y|Z=z)$ and $\sigma^{2}(z)=var(Y|T=t)$ are Lipschitz continuous, and bounded away from zero and infinity.

In the following, we only display the asymptotic results of ${\hat{\tilde{g}}}^{*}(z)$ and ${\hat{\tilde{g}}}_{e}^{*}(z;h_{5})$, where the asymptotic properties of ${\hat{\tilde{f}}}^{*}(t)$ and ${\hat{\tilde{f}}}_{e}^{*}(t;h_{6})$ can be obtained similarly.

**Theorem 1.** *Suppose that conditions A1–A4 hold, and bandwidth $h_{1}=\delta_{1}n^{-1/(2d+1)}$. Then we have*

$$n^{d/(2d+1)}\left[{\hat{\tilde{g}}}^{*}(z)-{\tilde{g}}^{*}(z)\right]\overset{D}{\to }N(b_{1}(z),v_{1}^{2}(z)),$$ (21)

where


$$b_{1}(z)=\frac{\delta_{1}^{d}}{d!}\left[\mu_{d}(K)D^{d}\tilde{g}(z)+\int \left\{\mu_{d}(K)\frac{m(z,t)f_{T}(t)}{f_{Z,T}(z,t)}D^{d}f_{Z,T}(z,t)-\mu_{d}(K_{2})D^{d}f_{T}(t)\right\}dt\right],$$

$$v_{1}^{2}(z)=\delta_{1}^{-1}||K|{|}_{2}^{2}\int \frac{f_{T}(t)}{f_{Z,T}(z,t)}\left\{\sigma^{2}(t)+m^{2}(z,t)\right\}dt$$

with $\sigma^{2}(t)=var(Y|T=t)$, $\mu_{d}(K)=\int u^{d}K(u)du$, *m*(*z*,*t*) is defined in Equation 8 and $||K|{|}_{2}^{2}=\int K^{2}(u)du$. $D^{d}g(z_{1},\ldots ,z_{q})=\sum_{j=1}^{q}\partial dg(z_{1},\ldots ,z_{q})/\partial z_{j}^{d}$.

If ${\hat{\tilde{g}}}^{*}(z)$ and ${\hat{\tilde{f}}}^{*}(t)$ in (18)-(20) are replaced by their true values, and $\bar{Y}$ is replaced by $\tilde{\alpha }$, then the estimators defined in (19) and (20) are referred to as *oracle estimators*, say ${\hat{\tilde{g}}}_{oracle}^{*}$ and ${\hat{\tilde{f}}}_{oracle}^{*}$, respectively. The asymptotic properties of the second-step estimators defined in (19) and (20) will be given below, implying that ${\hat{\tilde{g}}}_{e}^{*}(z)$ and ${\hat{\tilde{f}}}_{e}^{*}(t)$ behave asymptotically like the oracle estimators.

**Theorem 2.** *Suppose that conditions A1–A4 are satisfied, the bandwidth $h_{1}=o(n^{-1/(2d+1)}$, and $h=a_{0}n^{1/(2d+1)}$ for some a_0_>0. Then, for all ϵ, we have*

$$\mathrm{Pr}\left[n^{d/(2d+1)}\left\{{\hat{\tilde{g}}}_{e}^{*}(z;h,h_{e})-{\hat{\tilde{g}}}_{oracle}^{*}(z;h)\right\}>\epsilon |U^{n}\right]\to 0$$ (22)

with probability one, as n→∞, where $U^{n}=\{U_{1},\ldots ,U_{N}\}$ with $U_{i}=(Z_{i},T_{i}{)}^{⊤}$.

The proof of Theorem 1 and Theorem 5 directly follows that of kim *et al*. (1999) [21]. However, the covariate $z=x^{⊤}\hat{B}$ should be replaced by $\tilde{x}=x^{⊤}B$ in our setting. We will rewrite the asymptotic properties of ${\hat{\tilde{g}}}^{*}(z)$ as follows. The other asymptotic results can be obtained similarly.

**Theorem 3.** *Suppose that conditions A1–A4 hold, and bandwidth $h_{5}=\delta_{1}n^{-1/(2d+1)}$. Then we have*

$$n^{d/(2d+1)}\left[{\hat{\tilde{g}}}^{*}(z)-{\tilde{g}}^{*}(\tilde{x})\right]\overset{D}{\to }N(b_{2}(\tilde{x}),v_{2}^{2}(\tilde{x})),$$ (23)

where


$$b_{1}(\tilde{x})=\frac{\delta_{1}^{d}}{d!}\left[\mu_{d}(K)D^{d}\tilde{g}(\tilde{x})+\int \left\{\mu_{d}(K)\frac{m(\tilde{x},t)f_{T}(t)}{f_{\tilde{x},T}(\tilde{x},t)}D^{q}f_{\tilde{x},T}(\tilde{x},t)-\mu_{d}(K_{2})D^{d}f_{T}(t)\right\}dt\right],$$

$$v_{2}(\tilde{x})=\delta_{1}^{-1}||K|{|}_{2}^{2}\int \frac{f_{T}(t)}{f_{\tilde{x},T}(\tilde{x},t)}\left\{\sigma^{2}(t)+m^{2}(\tilde{x},t)\right\}dt+{\left[D{\tilde{g}}^{*}(\tilde{x})\right]}^{2}x^{⊤}var(\hat{B})x,$$

with $\sigma^{2}(t)=var(Y|T=t)$, $\mu_{d}(K)=\int u^{d}K(u)du$ and $||K|{|}_{2}^{2}=\int K^{2}(u)du$. $D^{d}g({\tilde{x}}_{1},\ldots ,{\tilde{x}}_{q})=\sum_{j=1}^{q}\partial dg({\tilde{x}}_{1},\ldots ,{\tilde{x}}_{q})/\partial {\tilde{x}}_{j}^{d}$, and $\hat{B}$ is the estimated basis direction in Section 3.1.

*Proof:* The proof can be completed directly from Theorem 1 and δ−method with the following fact:

$${\hat{\tilde{g}}}^{*}(z)\approx {\tilde{g}}^{*}(\tilde{x})+D{\tilde{g}}^{*}(\tilde{x})(z-\tilde{x})={\tilde{g}}^{*}(\tilde{x})+D{\tilde{g}}^{*}(\tilde{x})(x^{⊤}\hat{B}-x^{⊤}B).$$ (24)

◻

## Simulation Results

In this section, we consider two examples involving different regression functions to evaluate the finite sample performance of the proposed method. The model and parameter settings are as follows.

- (I) The structural dimension is *q* = 1, and $S_{Y|X}^{\left(T\right)}=\text{span}(\beta_{1})$, assuming that data are generated from the model:
  $$Y_{i}=\exp(X_{i}^{⊤}\beta )-\alpha +1.5\sin(2\pi T_{i})+\epsilon_{i},$$ (25)
- where constant α is given below, *X*_*i*_ is from the two scenarios (A) and (B) below, respectively, and *T*_*i*_ is independently generated from a uniform [0,1]. This implies that $E[g(X^{⊤}\beta )]=E[\exp(X^{⊤}\beta )-\alpha ]=0$ and E[f(T)]=E[1.5sin(2πT)]=0. The error $\epsilon_{i}\sim N(0,1)$ is independent and identically distributed. Additionally, assume that (*X*,*T*) is independent of ϵ. Now we consider the following cases about covariates to assess the linear condition for partial sliced inverse regression:

- (A) Covariate $X=(X_{1},X_{2},\ldots ,X_{20})$ is a 20-dimensional vector, where each component *X*_*k*_ is generated independently from a Bernoulli distribution with $\mathrm{Pr}(X_{k}=0)=\mathrm{Pr}(X_{k}=1)=0.5$. $\beta_{1}=\frac{1}{\sqrt{19}}(1,\ldots ,1,0{)}^{⊤}\in R^{20}$, implying that $\alpha ={\left[(\exp(\frac{1}{\sqrt{19}})-1)/2\right]}^{19}$.
- (B) We take $\beta_{1}=(1/\sqrt{2},0,1/\sqrt{2},0,\ldots ,0{)}^{⊤}\in R^{10}$, and covariate $X=(X_{1},X_{2},\ldots ,X_{10})$ is a 10-dimensional vector, where each component *X*_*k*_ follows a standard normal distribution *N*(0,1), and $\alpha =\exp(\frac{1}{2}).$

- (II) The structural dimension is *q* = 2; $\beta_{1}=(1/\sqrt{2},0,\ldots ,0{)}^{⊤}\in R^{10}$ and $\beta_{2}=(0,1/\sqrt{2},0,\ldots ,0{)}^{⊤}\in R^{10}$; $S_{Y|X}^{\left(T\right)}=\text{span}(\beta_{1},\beta_{2})$. We assume that the sample is from the model
  $$Y_{i}=1.5\sin(X_{i}^{⊤}\beta_{1})+\sin^{2}(X_{i}^{⊤}\beta_{2})-1+\sin(2\pi T_{i})+\epsilon_{i}.$$ (26)
  We keep all the other assumptions the same as in setting (I), but the covariates *X*_*i*_ and *T*_*i*_ are assumed to be correlated. Let $\tilde{W}=(X^{⊤}T{)}^{⊤}\in R^{p+1}$, where $\tilde{W}\sim N(0,\Sigma )$, with matrix elements Σ(j,j)=1 and Σ(i,k)=γ if i≠k. We consider the case of γ=0.2, which implies low correlation between covariates. Then, it is easy to find that $E[g(X^{⊤}\beta )]=E[1.5\sin(X^{⊤}\beta_{1})+\sin^{2}(X^{⊤}\beta_{2})-1]=0$ and E[f(T)]=0 with the standard normal distribution random variable $\tilde{W}$, since sin(2πt) is an odd function of *t*.

To assess the estimation accuracy of β using our proposed method, we take the rule of Fent *et al*. (2013) [18] by using the squared trace correlation coefficient [35], for the pair of generic random vectors $U=X^{⊤}\beta$ and $V=X^{⊤}\hat{\beta }$. The squared trace correlation coefficient is defined as $r^{2}=\text{tr}(A)/\text{dim}(A)$, where $A=\Sigma_{V}^{-1/2}\Sigma_{UV}\Sigma_{U}^{-1}\Sigma_{UV}\Sigma_{V}^{-1/2}$. Here, $\Sigma_{U}$ and $\Sigma_{V}$ are the variance matrices of *U* and *V*, respectively, and $\Sigma_{UV}$ is the covariance matrix between *U* and *V*. A squared trace correlation coefficient closer to unity indicates higher estimation efficiency. For further details, see Li and Dong (2009) [35] and references therein.

Table 1 illustrates the effectiveness of the proposed method in dimension reduction. It presents the means and the standard errors of ${\text{r}}^{2}$, computed from 1000 replicates for various sample sizes and slice numbers(K=5,10). The number of slices *K* refers to dividing the range of the response variable *Y* into *K* intervals (slices) of roughly equal size, with each slice used to estimate the relationship between *Y* and the covariates *X*. The slicing process is a key step in partial sliced inverse regression, helping to reduce data complexity while retaining essential statistical information. ${\text{r}}^{2}$ reflects the alignment between the true and estimated central subspaces, with values closer to 1 indicating better dimension reduction performance. The results demonstrate that higher sample sizes improve estimation accuracy, with ${\text{r}}^{2}$ values approaching unity. The proportion of correct structural dimension estimates by BIC is also examined in this simulation across 1000 repeated experiments for various scenarios. All experiments were conducted on a MacBook Air (M220222) equipped with an 8-core CPU, 8-core GPU, and 24GB of unified memory. We also recorded the time required for 1000 replicates. From the perspective of computation time, the proposed method exhibits reasonable computational performance.

**Table 1:** Mean and Standard Deviation (in parentheses) of *r*^2^ from 1000 Replicates for Models I and II

Combination	Sample size 4mm	Slice *K* = 5	Time (s)	Slice *K* = 10	Time (s)
(I,A)	n = 500	0.9805(0.0095)	62.47	0.9807(0.0092)	69.82
	n = 1000	0.9903(0.0045)	202.38	0.9903(0.0046)	232.44
	n = 2000	0.9901(0.0050)	$1.11\times 10^{3}$	0.9952(0.0023)	$1.32\times 10^{3}$
(I,B)	n = 500	0.9807(0.0091)	43.67	0.9882(0.0018)	52.19
	n = 1000	0.9895(0.0024)	128.11	0.9909(0.0041)	137.13
	n = 2000	0.9949(0.0017)	609.44	0.9950(0.0018)	623.38
(II,γ = 0.2)	n = 500	0.9082(0.0171)	$2.48\times 10^{3}$	0.9108(0.0235)	$2.67\times 10^{3}$
	n = 1000	0.9317(0.0043)	$9.65\times 10^{3}$	0.9419(0.0103)	$9.82\times 10^{3}$
	n = 2000	0.9589(0.0032)	$5.26\times 10^{4}$	0.9614(0.0073)	$5.57\times 10^{4}$
(II,γ = 0.4)	n = 500	0.9010(0.0208)	$2.54\times 10^{3}$	0.9085(0.0260)	$2.81\times 10^{3}$
	n = 1000	0.9303(0.0127)	$9.73\times 10^{3}$	0.9325(0.0115)	$1.04\times 10^{4}$
	n = 2000	0.9363(0.0132)	$5.38\times 10^{4}$	0.9409(0.0088)	$6.02\times 10^{4}$
(II,γ = 0.6)	n = 500	0.8405(0.0161)	$2.44\times 10^{3}$	0.8344(0.0140)	$2.96\times 10^{3}$
	n = 1000	0.8267(0.0106)	$9.86\times 10^{3}$	0.8133(0.0103)	$1.13\times 10^{4}$
	n = 2000	0.7917(0.0109)	$5.13\times 10^{4}$	0.7745(0.0122)	$6.11\times 10^{4}$

**Fig 1:**
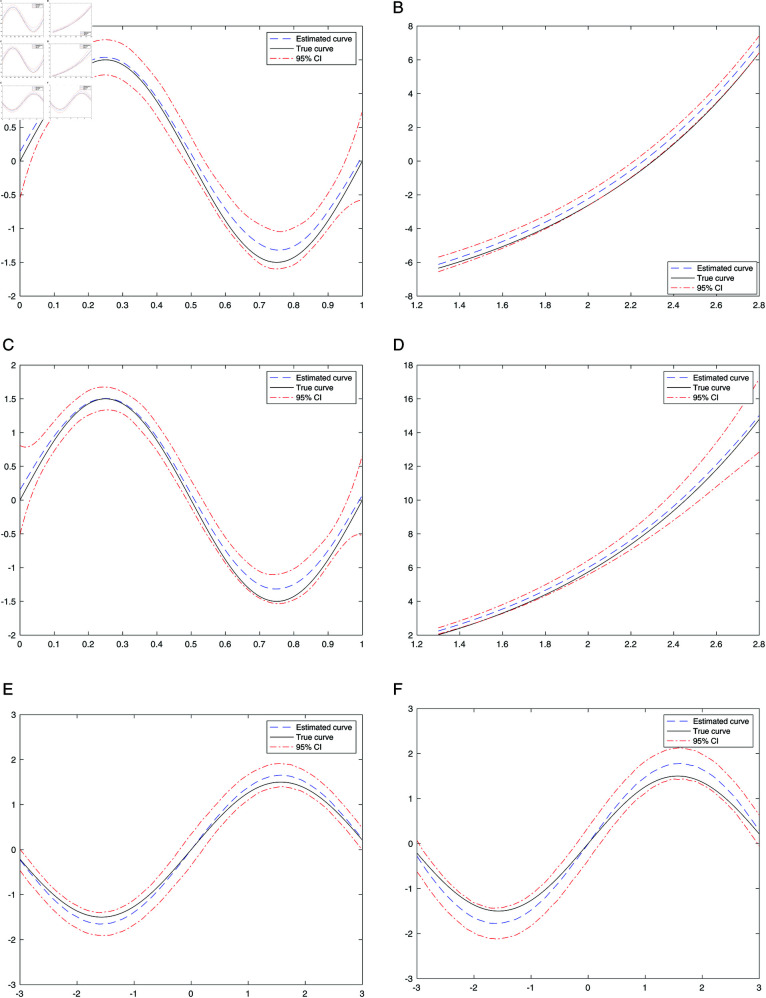
Estimated nonparametric components for simulation studies. a: Estimated *g*(*x*) for case (A, I). b: Estimated *f*(*t*) for case (A, I). c: Estimated *g*(*x*) for case (B, I). d: Estimated *f*(*t*) for case (B, I). e: Estimated *f*(*t*) for case II,γ = 0.2. f: Estimated *f*(*t*) for case II,γ = 0.6. *Note:* The nonparametric estimation plots are based on simulations with the sample size *n* = 1000 and the slice number *K* = 5 are chosen to reflect typical scenarios in dimension reduction tasks. In Case II, only f(t) is plotted to highlight the specific behavior of the response variable under these conditions. We present the estimation of f(t) for the weak correlation case (γ = 0.2) and the high correlation case (γ = 0.6).

Based on the obtained structural dimension and central subspace, fast instrumental variable pilot estimation is employed to estimate the nonparametric components $\tilde{g}(\cdot )$ and $\tilde{f}(\cdot )$. It is challenging to directly from the 3-dimensional picture for the estimated bivariate curve g(·) in a plane, and hence we only present the estimated curves of $\tilde{g}(\cdot )$ and $\tilde{f}(\cdot )$ of case *I* in the first four pictures of Figure 1, and f(·) for case (*II*) in the fifth picture of Figure 1.

From Table 1 and Fig 1 , it is evident that the estimated parameters and nonparametric components closely approximate their respective true values across the majority of conditions. Nonetheless, performance is subject to variation across different scenarios as a result of factors including covariate correlation, structural dimension, and sample size. As the correlation among covariates increases, the estimation accuracy for basis directions decreases, for instance, *r*^2^ drops from 0.9317 at γ=0.2 to 0.9303 at γ=0.4, and further to 0.8267 at γ=0.6 for *n* = 1000. This demonstrates that higher correlations among covariates can distort the estimation of the central subspace. Conversely, the method performs well under low correlation or independence. Larger sample sizes significantly enhance estimation precision. For instance, at γ=0.2, *r*^2^ increases from 0.9082 for *n* = 500 to 0.9589 for *n* = 2000 when *K* = 5. Similarly, for γ=0.4, *r*^2^ improves from 0.9010 for *n* = 500 to 0.9363 for *n* = 2000. However, in cases with high correlation as γ=0.6, the improvement is less significant, with *r*^2^ decreasing from 0.8405 for *n* = 500 to 0.7917 for *n* = 2000, indicating that high correlation poses challenges for accurate subspace estimation. The choice of slice numbers (*K* = 5 or *K* = 10) has a relatively minor impact on results, as *r*^2^ values remain consistent across most cases. For example, at γ=0.4 and *n* = 2000, the *r*^2^ values for *K* = 5 and *K* = 10 are 0.9363 and 0.9409, respectively. This suggests that the proposed method is robust to the choice of slice numbers, simplifying its practical implementation. Finally, the simulation results indicate that the proportion of correct structural dimension estimates using BIC is consistently 100% across all scenarios and sample sizes. This underscores the robustness of the proposed method in accurately determining structural dimensions, even under conditions with higher covariate dimensions and correlations. Comparing CaseI and CaseII, CaseII is slightly worse than CaseI due to the higher structural dimension (*d* = 2) and correlated covariates(γ=0.2,0.4,0.6). These factors increase the complexity of estimating the central subspace, leading to slightly reduced ${\text{r}}^{2}$ values.

- (III) We compare our proposed estimators with the ‘PLSI’ method by Xia and Härdle (2008) [36], which was also evaluated by Feng *et al*. (2013) [18], using the model they proposed:
  $$Y=\theta W+3\sin\left(\beta_{1}^{T}X/4\right)+0.2\varepsilon$$ (27)
  Here *X*,*W* and ϵ are independent and follow *N*(0,*I*_*p*_), *N*(0,1) and *N*(0,1), respectively; and θ=0.3. To investigate the effect of different dimensions of $\beta_{1}$, we consider two specific cases under Model III: Case III-A: The dimension of *X* is 6, ${\left(\begin{array}{c} 1/\sqrt{3},1/\sqrt{3},1/\sqrt{3},0,0,0 \end{array}\right)}^{T}$ and Case III-B: The dimension of *X* is 10 *p* = 10, ${\left(1/2,1/2,1/2,1/2,0,0,0,0,0\right)}^{T}$

Since the model and method proposed by Feng *et al*. (2013) [18] differ significantly from those presented in our paper, we make the following adjustments for the comparison. In our model, we treat the term $\left(\beta_{1}^{T}X/4\right)$ as g(·), while the component θW is considered as a whole and represented by f(·). Therefore, in the simulation study, we focus on comparing the performance of PLSI model for estimating $\beta_{1}$ and the whole part of θW. The sample size ranges from 500 to 2000, and the number of slices is fixed at *K* = 5. We report the squared trace correlation coefficient *r*^2^ between $\beta_{1}$ and its estimators.

**Fig 2:**
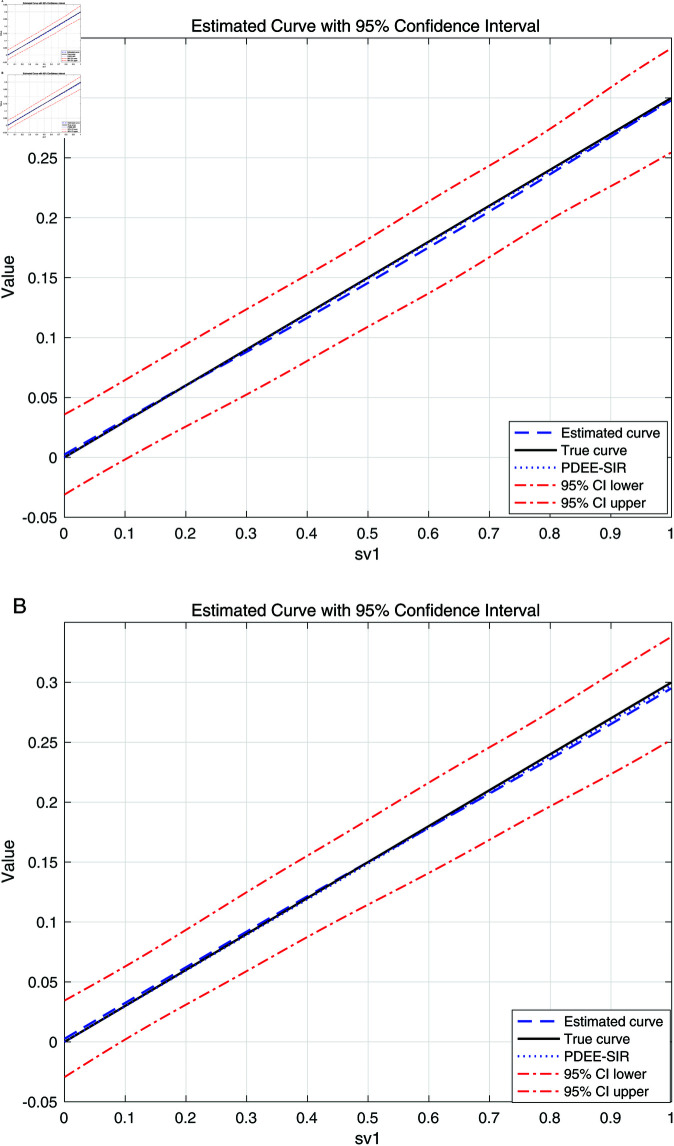
Comparison of Estimated θWb Curves: Proposed Method vs. PLSI a: case (III-A) with *n* = 1000. b: CaseIII-B with *n* = 1000.

As shown in Table 2, for estimating $\beta_{1}$ in the model, our method performs similarly to the PDEE-SIR method proposed by Feng *et al*. (2013) [18]. As shown in Figure 2, when treating θW as a whole, the resulting plot closely matches the one obtained by estimating θ first and then multiplying by W as done in Feng *et al*. (2013) [18], demonstrating the effectiveness of our estimation. Thus, for robustness, our method is recommendable, as it effectively handles the nonlinear component of our model, which poses identification challenges in the approach proposed by Feng *et al*. (2013) [18]. We also compared the computational efficiency of the two methods and found that our method is similar to the one proposed by Feng *et al*. (2013) [18], but slightly slower.

**Table 2:** Mean and Standard Deviation (in parentheses) of *r*^2^ from 1000 Replicates for Model III with Different Methods

Case	Sample size	method 1 (Feng *et al*., 2013)	Time(s)	Method 2 (Proposed Method)	Time(s)
III-A	500	0.9893 (0.0073)	41.34	0.9984 (0.0007)	53.98
III-A	1000	0.9905 (0.0048)	154.56	0.9995 (0.0008)	163.56
III-A	2000	0.9941 (0.0048)	452.57	0.9949 (0.0004)	523.71
III-B	500	0.9875 (0.0050)	52.46	0.9962 (0.0020)	61.17
III-B	1000	0.9901 (0.0065)	162.79	0.9981 (0.0011)	185.34
III-B	2000	0.9937 (0.0055)	593.23	0.9992 (0.0002)	671.89

## Application

The Medical Expenditure Panel Survey (MEPS) is a nationally representative survey of healthcare utilization and expenditures for the civilian non-institutionalized population in the United States [37]. This study analyzes a subset from the 2010 Full Year Consolidated Data File of the Household Component, which includes detailed information on health conditions, medical expenditures, insurance coverage, and demographic characteristics.

This article examines medical data from elderly households surveyed by MEPS in 2010, where all members were aged 65 or older. The dataset includes a total of 2139 individuals aged between 65 and 84 years. Chen *et al*. (2014) [7]analyzed this dataset using a partial prior marginal model and highlighted that medical expense data is often highly skewed to the right, with a large mean $9235 but a small median $3955.

Among the sample, 41.1% of males had hospitalization experience, with a mortality rate of 15.0%. Additionally, 79.5% of white individuals with hospitalization experience had a mortality rate of 1.4%. Key medical conditions such as cardiovascular diseases (82.7%), physical dyskinesia (76.6%), cancer (28.9%), diabetes (21.9%), and respiratory diseases (16.9%) were found to significantly influence medical expenses. Based on these findings, Chen *et al*. (2014) [7] selected these covariates for their analysis.

However, MEPS data contains a vast number of covariates, and selecting only a subset of them may not provide a comprehensive understanding of medical expenses. To address this, this study applies the partially nonlinear index model proposed earlier to analyze MEPS data. This model not only accommodates high-dimensional covariates but also captures the nonlinear effects of time-related covariates on medical expenses.

In addition to age, which is modeled as a continuous covariate, the following 14 variables are also included in the analysis:

- **White Race (*X***_**1**_**):** *X*_1_ = 1 for white individuals, *X*_1_ = 0 for non-white individuals.
- **Gender (*X***_**2**_**):** *X*_2_ = 1 for male, *X*_2_ = 0 for female.
- **Mortality (*X***_**3**_**):** *X*_3_ = 1 if deceased, *X*_3_ = 0 otherwise.
- **Hospitalization (*X***_**4**_**):** *X*_4_ = 1 if hospitalized, *X*_4_ = 0 otherwise.
- **Cardiovascular Diseases (HBV Disease, *X***_**5**_**):** *X*_5_ = 1 if diagnosed, *X*_5_ = 0 otherwise.
- **Respiratory Diseases (*X***_**6**_**):** *X*_6_ = 1 if diagnosed, *X*_6_ = 0 otherwise.
- **Body Movement Disorders (*X***_**7**_**):** *X*_7_ = 1 if diagnosed, *X*_7_ = 0 otherwise.
- **Cancer (*X***_**8**_**):** *X*_8_ = 1 if diagnosed, *X*_8_ = 0 otherwise.
- **Diabetes (*X***_**9**_**):** *X*_9_ = 1 if diagnosed, *X*_9_ = 0 otherwise.
- **Family Size (*X***_**10**_**):** *X*_10_ = 1 for households with at least two members, *X*_10_ = 0 for individuals living alone.
- **Supplemental Insurance (*X***_**11**_**):** *X*_11_ = 1 if the individual has additional insurance apart from Medicare, *X*_11_ = 0 otherwise.
- **Family Income Levels (*X***_**12**_**):** *X*_12_ = 1 for poor, *X*_12_ = 2 for near poor, *X*_12_ = 3 for low income, *X*_12_ = 4 for middle income, and *X*_12_ = 5 for high income.
- **Medicare Coverage (*X***_**13**_**):** *X*_13_ = 1 if covered by Medicare, *X*_13_ = 0 otherwise.
- **Years of Education (*X***_**14**_**):** *X*_14_ = 0 for no education, *X*_14_ = 1 to 8 for elementary education, *X*_14_ = 9 to 12 for high school education, *X*_14_ = 13 to 16 for college education, and *X*_14_ = 17 or higher for postgraduate education.

For convenience, in the process of partial sufficient reduction, we use the standardized variable *X* to obtain the basis direction $\tilde{B}$, and then the true basis direction *B* is defined as $B=\Sigma_{X}^{1/2}\tilde{B}$, where $\Sigma_{X}$ is the covariance matrix of *X*. The estimated basis direction *B*, based on the partially sufficient dimension reduction with *K* = 5, is displayed in Table 2. The estimated structural dimension *q* = 2.

From Table 3, we can see that hospitalization is largely reflected in both central subspace directions, while heart and blood vessel disease, body movement disorder, having additional insurance, and having Medicare coverage are only reflected in the second direction. Hospitalization plays the most influential role in the first central subspace directions, and heart and blood vessel disease plays the most influential role in the second central subspace directions. The important factors or variables are correctly identified, and the results are consistent with those of Chen *et al*. (2016) [4].

**Table 3:** Partially sufficient dimension reduction for MEPS data.

Covariates 14mm	1st direction	2nd direction
White Race	0.0049	0.0638
Male	-0.0269	-0.0872
Mortality	0.0921	-0.1158
Hospitalization	0.9173	-0.3716
HBV Disease	0.1631	0.4705
Respiratory Disease	0.1602	0.0455
Body Movement Disorder	0.1050	0.2368
Cancer	0.1182	0.1021
Diabetes	0.1650	0.1818
Family size	-0.0330	-0.0780
Supplemental Insurance	-0.1860	-0.5965
Family Income Levels	0.0188	0.0223
Medicare Coverage	0.0941	0.3884
Years of Education	0.0027	0.0228

**Fig 3:**
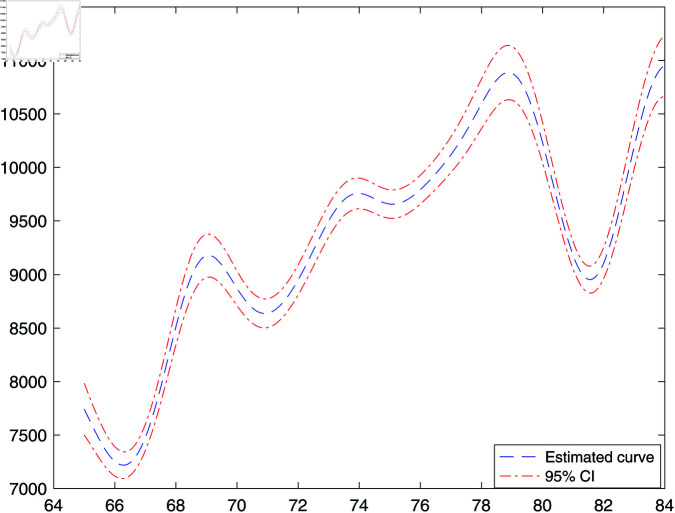
Estimated f(t) for MEPS data.

After getting the estimated structural dimension and the basis direction, a standard additive model can be established. The estimated nonlinear age effect in f(·) is shown in Figure 3. We can see that there is a small dip at first, then the age effect increases from 66 to 69. There are some fluctuations between 69 and 79, followed by a significant dip at 82. After reaching age 82, the medical cost exhibits a clearly increasing pattern. These nonlinear age effects reveal important age-specific variations in medical costs, offering insights into healthcare policy. The significant increase in costs from ages 66 to 69 indicates the necessity for targeted preventive care and chronic disease management to address early-onset risks in this demographic. The fluctuating trends between 69 and 79 emphasize the importance of personalized healthcare strategies to address diverse needs in this age group, ensuring resources are allocated effectively. Meanwhile, the steep increase after age 82 underscores the necessity of robust elderly care systems, including long-term care insurance and community-based support services, to manage the escalating financial burden on healthcare systems. These findings align with those of Chen *et al*. (2016) [4], further validating the proposed model’s capacity to uncover meaningful patterns in medical cost data.

## Concluding Remarks

This paper proposes a partially nonlinear index model (PNIM) that effectively addresses the challenges of high-dimensional covariates and nonlinear covariate effects. The partially sufficient dimension reduction framework estimates structural dimensions and basis directions, providing computational efficiency and robustness. Next, Using the fast instrumental variable pilot estimation, the nonparametric components were estimated within the standard additive model framework. We also established the asymptotic properties of the estimators. The simulation results and the analysis of the MEPS dataset demonstrated the model’s practical utility, particularly in identifying key factors that influence medical costs and their nonlinear relationships.

Our analysis of the MEPS data uncovered several significant findings that could inform policy decisions. Hospitalization was identified as the most influential factor in the first central subspace direction, highlighting its crucial role in determining medical costs. This indicates that policy interventions focused on reducing avoidable hospital admissions—such as enhancing outpatient care, preventive health programs, and post-discharge management—could greatly ease the financial burden on the healthcare system. The nonlinear age effects, as illustrated by the regression curve, highlight critical age-specific variations in medical costs. The significant rise in costs between ages 66 and 69 highlights the necessity for targeted chronic disease prevention programs for this demographic. In contrast, the swift increase in costs after age 82 necessitates enhanced long-term care policies, which include better access to long-term care insurance and community-based support systems for seniors. It also highlights the role of chronic conditions and supplemental insurance. Variables such as heart disease, blood vessel disease, and supplemental insurance coverage significantly influenced the second central subspace direction. These findings highlight the importance of policies that emphasize targeted prevention and management of chronic diseases while ensuring access to affordable supplemental insurance for at-risk populations. These results highlight the potential of PNIM as a strong analytical framework for understanding the complex interplay of factors influencing medical costs, offering actionable insights to guide evidence-based healthcare policy.

Future research will address some limitations of the current framework. One notable challenge is that medical cost data often exhibit inherent skewness (long tails to the right) and heteroscedasticity (variance that changes across different levels of covariates), which may not be fully captured by the current model’s emphasis on mean regression and presumption of homoscedasticity [7]. Skewness can lead to biased estimates when the mean is dominated by extreme values, while heteroscedasticity can affect the efficiency and consistency of parameter estimates. These characteristics are particularly prominent in medical cost data due to the high variability in healthcare utilization among different demographic and socioeconomic groups.

A promising direction involves extending the partially sufficient dimension reduction framework to account for these distributional properties. Specifically, adopting the MAVE approach [24] within the framework of quantile regression, as suggested by Kong and Xia (2014) [38], could allow for a more robust analysis. Quantile regression not only addresses skewness by focusing on conditional quantiles rather than the mean but also naturally accommodates heteroscedasticity by estimating covariate effects across the entire distribution of the response variable. This would enable a more comprehensive understanding of cost drivers across different percentiles, such as the lower quantiles (representing low-cost groups) and upper quantiles (representing high-cost outliers).

By analyzing the entire distribution of medical costs, this extension would provide deeper insights into the varying effects of covariates on healthcare expenditures. For example, certain predictors may have a greater impact on high-cost outliers than on average patients, which has significant implications for designing targeted healthcare policies. This approach would enhance the utility of the model in addressing the complexities of medical cost data and guide the development of evidence-based policies that account for variability across population subgroups.

Additionally, another limitation is that our current model assumes complete observations and does not explicitly address missing data issues, which are common in real-world medical datasets. Standard sufficient dimension reduction methods, including our proposed approach, generally require complete data, limiting their applicability in the presence of missing values. Future extensions could incorporate methods such as multiple imputations or inverse probability weighting to effectively handle missing data. Recent studies, such as Xue and Zhang (2020) [39], have developed empirical likelihood-based approaches for partially linear single-index models with missing observations, demonstrating how bias correction and imputation techniques can enhance model robustness. In the future, we can refer to Xue and Zhang’s (2020) [39] expansion of empirical likelihood in our model to better adapt to scenarios with missing data and enhance the model’s applicability in empirical medical research.
